# Challenges to current medical education and practice

**DOI:** 10.4103/0970-0358.41123

**Published:** 2008

**Authors:** Anil Nirale

**Affiliations:** Consultant Plastic and Cosmetic Surgeon, www.icls.in, SV Patel Road, Borivali (W), Mumbai, India

Dear Sir,

There are changes happening at every level in the medical field and medical education is no exception. As a multitude of factors play increasingly important roles in the execution of the medical knowledge acquired during one′s education, it is high time that medical colleges themselves adopted radical changes in their curriculum.

## Changes in patients' expectations

As a result of the move to a more open society with ready access to medical information *via* the Internet and other media, patients have seemingly become ‘wise’ and ‘well-informed’ about health, disease and treatment options.

## Change in healthcare delivery

The style of practice has changed for most doctors. Multispeciality hospital practices have replaced the individual ‘doctor-patient’ relationship perhaps compromising patient care to some extent.

The emergence of “Third party Administrators” (TPAs), which has changed medical insurance policies, has also begun to show its effect on the medical profession.

## Changes in medical knowledge

New subjects and material have certainly been added to curricula but there has been a reluctance to remove older topics, possibly limiting the ability of the course to develop as expected.

## Suggestion

1. All practical topics should not be repeated in theory classes.
2. The importance of certain topics in the contemporary practice of medicine must be re-evaluated. Lectures on these topics should be completely cancelled and these topics or subjects, while still part of the syllabus, should be set aside for self-study. This way, the precious time set aside for lectures can be utilized to cover other more important as well as newer topics.

## Changes in students' requirements

It has been aptly said that currently medical courses seem not only to contribute to the disillusionment and demoralization of students by deadening their initial enthusiasm for medicine but also fail to prepare them adequately for the diversity of problems, which they will encounter as practicing professionals. Graduates are also always under pressure to recover the cost incurred in their medical education as early as possible, sometimes leading to a loosening of ethical standards.

## Suggestion

1. Let students earn while they learn.
2. Let us devise a points' system and let them work in wards right from their 1^st^ year of MBBS onwards. We are always running short of staff in hospitals. Students try to find other part-time avenues to earn money. Why can't we provide them better, more professionally relevant alternatives and also meet our own staffing needs in the process?

There are many activities going on in hospitals in various departments for which medical students can be hired instead of assigning these tasks to untrained/non-medical hospital staff. Medical students can do dressings and other clerkship procedures and earn points for these and get paid according to the points they earn.

## Where do we go next?

As we take a fresh look at current trends in medical education, we can see several key issues which can be improved by introducing:

- Outcome-based education
- The introduction of new learning technologies
- The introduction of new courses
- Choice of educational strategies
- Staff development and professionalism in medical education

## Outcome-based education - What sort of a doctor is needed?

“Outcome-based education” means an education, which will produce a doctor who is fully competent and prepared to start a basic medical practice by the time of graduation. A medical graduate should not be wasting time in learning new “tricks” after putting in 51/2-6 years into graduation.

At this stage, medical curricula can be extended by a year if necessary but the outgoing doctor must be well-trained in all practical aspects.

The goal should be to have medical graduates who have less knowledge but more abilities than current graduates. These abilities will include appropriate attitudes, problem-solving skills and the use of information technology. Doctors must have an appreciation of a wider variety of possible solutions to healthcare problems including complementary and alternative medicine.

They must participate in Rural health development projects (actual ‘brick level’ project work must be done by medical graduates). Three month terms in ayurvedic, homeopathy and alternative medicine including yoga, reiki and holistic methods would also help.

New learning technologies - what can be done differently?

Enormous advances have taken place in information technology (IT), which can also be added to curricula as medical training tools.

Topics which can be added include:

- IT
- Computers
- Medical transcription
- English-speaking and writing, personality development
- Marketing, business management
- Hospital administration
- Medical insurance
- Clinical research, drug trials, etc.

## The introduction of new courses

The time is ripe to introduce new courses, which will be more “end user-friendly” rather than just being academic. Communication skills, preparation for practice and evidence-based practice must find a place in revised curricula.

## Choice of educational approach - What is the best strategy?

There is no single “best” curriculum. There should be more flexible learning opportunities with an adaptive curriculum in which the different learning needs of individual students are recognized and the program could perhaps be tailored to meet these needs.

## Staff development - Can excellence in teaching be fostered?

“Like teacher, like student.” Both credit and blame go to the teacher. It is the teacher who imparts skills or knowledge to others. Hence, it is important to first prepare good teachers. What is a good teacher?

A good teacher is one who teaches NOT by words but by actions.

Teachers will be respected and their lectures attended only if students are convinced about the genuine abilities of the teachers to impart their skills on to the students. Just like patients can discern which doctors are good and which are not, students too can see which teachers are capable and which are not.

Just increasing the remuneration of teachers will NOT help. The performance and dedication of teachers like doctors cannot be improved or heightened by more money. Increasing pay can certainly make them feel more secure financially so that they can devote their attention to teaching. However they must also be made to genuinely feel that they are one of the most important cogs in the wheel of medical education.

As is the practice followed in Armed Forces Medical College special incentives can perhaps be offered to teachers in all departments depending on the performance of their students.

Regular teacher training programs will also surely help.

A “reverse” assessment by *students* of their teachers should be done every three months, which should be taken into consideration during the promotions of the teachers.

Research and publications should be compulsory for teachers to get promotion. In fact, there is a rule that any organization registered as a ‘research’ society has to produce research papers.

Inviting competent persons from private non-teaching institutions as temporary faculty should be encouraged.

Instead of forcing students to attend lectures, an environment should be created in such a way that they should be eager not to miss a single lecture.

An amateur role for a medical teacher is no longer sustainable. Competence in teaching should be a requirement for all teachers.

Only when one analyzes the data in its entirety can one start to appreciate the existence and the magnitude of a problem. I end by saying that medical education is not an exception to this.

